# A postnatal role for embryonic myosin revealed by MYH3 mutations that alter TGFβ signaling and cause autosomal dominant spondylocarpotarsal synostosis

**DOI:** 10.1038/srep41803

**Published:** 2017-02-16

**Authors:** Jennifer Zieba, Wenjuan Zhang, Jessica X. Chong, Kimberly N. Forlenza, Jorge H. Martin, Kelly Heard, Dorothy K. Grange, Merlin G. Butler, Tjitske Kleefstra, Ralph S. Lachman, Deborah Nickerson, Michael Regnier, Daniel H. Cohn, Michael Bamshad, Deborah Krakow

**Affiliations:** 1 Department of Human Genetics, David Geffen School of Medicine at UCLA, California, 90095, USA; 2 Department of Molecular, Cell and Developmental Biology, University of California Los Angeles, Los Angeles, California, 90095, USA; 3 Department of Genome Sciences, University of Washington, Seattle, Washington 98195, USA; 4 Division of Genetic Medicine, Department of Pediatrics, University of Washington, Seattle, WA, 98195, USA; 5 Division of Genetic Medicine, Seattle Children’s Hospital, Seattle, WA 98105, USA; 6 Department of Orthopaedic Surgery, David Geffen School of Medicine at UCLA, Los Angeles, California, 90095, USA; 7 Division of Genetics and Genomic Medicine, Department of Pediatrics, Washington University School of Medicine, Saint Louis, Missouri, USA; 8 Departments of Psychiatry & Behavioral Sciences and Pediatrics, University of Kansas Medical Center, Kansas City, KS, USA; 9 Department of Human Genetics, Radboud University Medical Center, Nijmegen, The Netherlands; 10 International Skeletal Dysplasia Registry, University of California Los Angeles, Los Angeles, California, 90095, USA; 11 Department of Bioengineering, University of Washington, Seattle, WA, USA; 12 Institute for Stem Cell and Regenerative Medicine, University of Washington, Seattle, WA, USA; 13 Center for Cardiovascular Biology, University of Washington, Seattle, WA, USA; 14 Orthopaedic Institute for Children, University of California Los Angeles, Los Angeles, California, 90095, USA; 15 Department of Obstetrics and Gynecology, David Geffen School of Medicine at UCLA, Los Angeles, California, 90095, USA.

## Abstract

Spondylocarpotarsal synostosis (SCT) is a skeletal disorder characterized by progressive vertebral, carpal and tarsal fusions, and mild short stature. The majority of affected individuals have an autosomal recessive form of SCT and are homozygous or compound heterozygous for nonsense mutations in the gene that encodes the cytoskeletal protein filamin B (FLNB), but a subset do not have *FLNB* mutations. Exome sequence analysis of three SCT patients negative for *FLNB* mutations identified an autosomal dominant form of the disease due to heterozygosity for missense or nonsense mutations in *MYH3*, which encodes embryonic myosin. Cells transfected with the *MYH3* missense mutations had reduced TGFβ signaling, revealing a regulatory role for embryonic myosin in the TGFβ signaling pathway. In wild-type mice, there was persistent postnatal expression of embryonic myosin in the small muscles joining the neural arches of the spine suggesting that loss of myosin function in these muscles contribute to the disease. Our findings demonstrate that dominant mutations in *MYH3* underlie autosomal dominant SCT, identify a postnatal role for embryonic myosin and suggest that altered regulation of signal transduction in the muscles within the spine may lead to the development of vertebral fusions.

Spondylocarpotarsal synostosis (SCT) is a skeletal disorder characterized by progressive fusions of the vertebral bodies, carpal and tarsal coalitions, delayed endochondral ossification, and mild short stature^1^. The majority of affected individuals have an autosomal recessive form of SCT and are homozygous or compound heterozygous for loss-of-function mutations in the gene that encodes filamin B (*FLNB*, MIM 603381)^2^. However, an autosomal dominant form of SCT (AD SCT) has also been described in an affected mother and son in whom mutations in both *FLNB* and *Noggin* were excluded^3^, indicating that there is locus heterogeneity in SCT.

*FLNB*, the first identified gene associated with SCT, encodes a cytoskeletal protein that functions as a stabilizer for actin cytoskeletal networks as well as an integrator of cellular signaling (reviewed in ref. 4). In a *Flnb* knockout mouse model of SCT, we showed that progressive vertebral fusions occur through early degeneration, collapse, and eventual mineralization of the intervertebral disc (IVD)^5^. The vertebral fusions resulted in part from up regulation of both the Transforming Growth Factor β (TGFβ) and Bone Morphogenetic Protein (BMP) signaling pathways within the IVD^5^.

All methods utilized were approved under a University of California at Los Angeles human subjects protocol and all subjects provided informed consent. We studied three unrelated SCT families (International Skeletal Dysplasia Registry reference numbers R12-336, R07-183B and R06-109) in whom mutations were not identified in *FLNB*. To identify the gene underlying SCT in these families, exome sequencing and analysis in accordance with approved guidelines at the University of Washington Center for Mendelian Genomics as previously described^6^. Briefly, the exome sequencing library was prepared with the NimbleGen SeqCap EZ Exome Library v2.0 kit and sequenced on the Illumina GAIIx platform. Reads were mapped with BWA^7^ to the human reference genome (hg19/GRCh37) and duplicated reads were marked with Picard (http://picard.sourceforge.net). Variants were called using the Genome Analysis Toolkit following their Best Practices recommendations and were annotated with VEP version 83^8^. The data were first reviewed for potentially causative variants in *FLNB* and the gene was again excluded. Because SCT appeared to be transmitted in a dominant pattern in family R07-183 (Supplemental Fig. 1C), all heterozygous non-synonymous substitutions and variants affecting splice junction consensus sequences, all with a global frequency of <1% in the Exome Sequencing Project (ESP)^9^ and the Exome Aggregation Consortium (ExAC)^10^ databases were considered candidates. We analyzed the data to determine if multiple individuals in the cohort had changes in the same gene. Furthermore, family segregation analysis was performed for family R07-183 where variants present in the unaffected parents were excluded.

Affected individuals from all three families were heterozygous for variants in *MYH3*, which encodes embryonic myosin heavy chain 3 (Table 1). Individual R06-109A was heterozygous for a *de novo* variant, c.1934T > G, predicted to result in a p.Phe645Cys substitution (Supplemental Fig. 1). Individual R07-183B, from a family with a three-generation history of SCT, was heterozygous for a 3 base pair in-frame deletion, c.272-729delTCC, predicted to result in deletion of serine 243 [(p.Ser243del), Supplemental Fig.1]. The third affected individual, R12-336, was heterozygous for a frameshift deletion, c.2699delT (p.Leu900fs9), predicted to lead to a premature termination codon (Supplemental Fig. 1). Collectively, these results suggest that mutations in *MYH3* underlie AD SCT.

The clinical and radiographic characteristics for each affected individual with an *MYH3* mutation are summarized in Fig. 1 and Table 2, and are similar to those of individuals with SCT resulting from loss-of-function mutations in *FLNB*. There were progressive vertebral fusions in the cervical, thoracic, lumbar and sacral regions of the spine as well as carpal and tarsal coalitions. Radiographs (Fig. 1) demonstrate the progressive nature of the vertebral fusions, ranging from mild disc space narrowing in a three-year-old to vertebral fusions and severe scoliosis in an affected adult. All three individuals had bilateral fifth finger clinodactyly, one (R06-109) had a cleft palate, and one (R07-183B) had mild hand contractures with syndactyly, elbow and knee dislocations, and had surgery as a child to correct overlapping fifth toes (Table 2). R07-183B is a member of a large family with autosomal dominant SCT (Supplemental Fig. 1C). Most affected individuals in this family reported only scoliosis and vertebral fusions, some had mild hand contractures, and the affected offspring of the proband had a cleft palate. None of the affected individuals were reported to have congenital pterygium, severe campodactyly, or equinovarus deformities (Table 2).

Embryonic myosin is a class II myosin molecule that consists of head, neck, and tail domains and functions in muscle contraction by binding to and exerting force on actin filaments through the hydrolysis of ATP^11^. The p.Phe645Cys substitution resides in the outer region of the myosin head domain, which contains the myosin motor that allows the molecule to exert force on actin fibers during muscle contraction (Supplemental Fig. 2). The substitution resides in loop 2 of myosin, which interacts directly with actin and is responsible for initial, weak binding of myosin to actin and thus is critical for normal myosin function. Presence of an unpaired cysteine may allow for disulfide bonding with an intramolecular cysteine, such as the cysteine residue at residue 539, possibly changing the overall conformation of the head domain and affecting its ability exert force on the actin network. The p.Ser243del mutation lies near the nucleotide-binding domain, a domain that is essential to the binding of ATP during muscle contraction (Supplemental Fig. 2). A mutation at this site could alter the geometry surrounding the nucleotide-binding site and reduce the ability of embryonic myosin to exert force on the actin network. The frameshift mutation predicts a premature stop codon nine codons downstream (Supplemental Fig. 1B). The ExAC database reports *MYH3* alleles harboring nonsense mutations with very low allele frequencies, but no phenotypic information is available, so the clinical consequence of these variants is unclear. It is possible that nonsense mutations within different domains of the molecule may have varying effects. Since affected individual R12-336 was ascertained in a cohort of *FLNB* negative SCT patients, two of whom had mutations in *MYH3*, we interpret the nonsense allele for embryonic myosin as likely to have phenotypic consequences.

To determine whether the *MYH3* mutations lead to synthesis of stable proteins, we used a *MYH3*-GFP fusion plasmid (Origene, cat#RG218098) and performed site-directed mutagenesis (QuikChange II site-directed mutagenesis kit, Agilent, 200521) to introduce each mutation. Following confirmation by Sanger sequence analyses (Supplemental Fig. 3), the mutated plasmids were transfected into human embryonic kidney (HEK) cells using the Lipofectamine 3000 Reagent Kit (ThermoFisher, L3000015). HEK cells have been shown to express a low level of endogenous *MYH3*^12^ (Fig. 3A). Cells were grown in DMEM with 10% FBS and then lysed using RIPA buffer. Protein samples derived from the transfected cells were then analyzed by Western blotting. Plasmids harboring the p.Phe645Cys and p.Ser243del substitutions both produced full length proteins, extended by the GFP tag, when probed with embryonic myosin antibody (Fig. 3A). However, the p.Ser243del protein was less stable when compared to WT MYH3 (p = 0.003) and the p.Phe645Cys protein (p = 0.01) (Fig. 3C). The plasmid harboring c.2699delT produced a truncated protein of about 100 kDa (Fig. 3A, arrow), the size predicted based on the location of the nonsense mutation.

To further explore the pathophysiology of the vertebral fusions in SCT caused by defective embryonic myosin, we defined the expression pattern of embryonic myosin in the spine. Wild-type mouse spine sagittal sections at embryonic day 15.5 (E15.5), postnatal day 1 (P1), and P15 were stained using an antibody against embryonic myosin (Genetex, #GTX32147) and DAB chromogen. Immunohistochemical staining confirmed that embryonic myosin is expressed in bone (Fig. 2A)^13^,^14^ and further revealed high expression in the small muscles that attach at the distal neural arches of the spine at E15.5, P1, and P15 in both the cervical and thoracic regions (Fig. 2A, arrows). These small multifidus muscles fill the grooves on either side of the spinous processes of the vertebrae from the axis to the sacrum and play an important role in stabilizing the joints between the vertebral bodies^15^. While embryonic myosin expression was strongest in E15.5 and P1 mice, it persisted at slightly lower levels at P15. These findings indicate that postnatal embryonic myosin expression persists in the region of the spine that is affected in SCT. There was no expression of embryonic myosin within the annulus fibrosus or the nucleus pulposus of the IVD at any age, an expression pattern distinct from FLNB, which is expressed in these regions^5^,^16^. We confirmed these findings by RT-PCR using annulus fibrosis and nucleus pulposus RNA derived from P15 mouse IVDs using primers for *Myh3* cDNA (Fig. 2B). Thus, the specificity and ongoing expression of embryonic myosin in the small multifidus muscles connecting to the neural arches is correlated with the phenotypic findings in autosomal dominant *MYH3* SCT, suggesting a role for these muscles in regulating forces surrounding the IVD. Because the patients have mild short we also interrogated wild type P15 mouse cartilage growth plates to explore if MYH3 is expressed in growth plate chondrocytes. We did not detect expression of MYH3 in chondrocytes (data not shown)^13^.

In our earlier study on SCT using a *Flnb*^−/−^ mouse model, loss of FLNB produced changes in TGFβ/BMP signaling cascades in the IVD^5^. In TGFβ /BMP signaling, the respective ligands bind to and/or bring together different combinations of type I and type II serine/threonine kinase receptors at the cell surface. Ligand binding to the type II receptor produces a phosphorylation cascade from the type II to the type I receptor, which then propagates the signal via receptor Smad proteins^17^ as well as non-canonical signaling through extracellular signal-related kinase (ERK), c-Jun N-terminal kinase (JNK), and p38 mitogen activated protein (MAP) kinase. Using transfected HEK cells, we sought to determine whether the *MYH3* missense mutations affected TGFβ signaling. After transfection with the wild-type (WT) or mutated plasmids, HEK cells were serum starved for two hours and then stimulated with TGFβ1 (5 ng/mL). The cells were lysed using RIPA buffer and the proteins analyzed by Western blotting. Western blots with TGFβ1-stimulated cell proteins were probed with p-Smad3 antibody, as Smad3 has historically been shown to be more important in muscle development than Smad2^18^. Membranes were also probed with antibodies against p-ERK1/2 and p-p38 to analyze the non-canonical components of the pathway. Protein band intensities were quantified using ImageJ software and normalized to both GAPDH as well as the amount of transfected MYH3 protein in each sample. Statistical analyses were performed by Student’s T-test, comparing the non-transfected and mutated plasmid effects to WT plasmid effects.

Transfection of WT embryonic myosin into HEK cells resulted in increased Smad3 (p = 0.02) and p38 phosphorylation (p = 0.009) relative to the negative control plasmid, indicating that the presence of MYH3 had an overall stimulatory effect on TGFβ signaling. Relative to WT MYH3 there was a decrease in Smad3 phosphorylation in cells transfected with the p.Phe645Cys, p.Ser243del, and p.Leu900fs9 mutant plasmids (p = 0.03, p = 0.03, and p = 0.05, respectively) (Fig. 3B,D). No change in ERK phosphorylation levels was seen with TGFβ1 stimulation, however there was a significant decrease in p38 phosphorylation (p = 0.01) in cells transfected with the p.Leu900fs9 mutant plasmid and a trend toward decreased levels in cells expressing the p.Phe645Cys and p.Ser243del proteins (p = 0.07 and p = 0.07, respectively) (Fig. 3B,E,F). Overall, p.Phe645Cys, p.Ser243del and p.Leu900fs9 expression resulted in decreased activation of both canonical and noncanonical TGFβ signaling when compared to WT. These results demonstrate that mutated MYH3 can have an inhibitory effect on the TGFβ pathway. Inhibition of TGFβ signaling can result in muscle cell hypertrophy (reviewed in ref. 19) and previous work in cultured skeletal muscle cells with the p.R672C embryonic myosin substitution found that myofibers were larger in diameter compared to controls, had reduced specific force, a prolonged time to relaxation and incomplete relaxation^20^. Our results show that the missense and nonsense mutations in embryonic myosin negatively impacted the tightly regulated TGFβ pathway, key to muscle function, and may affect phenotype by inducing inappropriate muscle hypertrophy and function.

Mutations in *MYH3* have been previously shown to underlie distal arthrogryposis type 2A (DA2A; Freeman-Sheldon syndrome [MIM 193700]), DA2B (Sheldon-Hall syndrome [MIM 601680]) and DA8 (AD multiple pterygium syndrome [MIM 160720])^13^,^21^. DA2A is characterized by congenital contractures of the upper and lower limbs, distinctive facial features, and motor delays. The phenotypic expressivity is highly variable^22^. DA2B is the most common of the distal arthrogryposis syndromes^23^ and the congenital contractures tend to be similar though milder than DA2A. DA8, or autosomal dominant multiple ptyergium syndrome, is characterized by congenital contractures, multiple pterygia, scoliosis, hemivertebrae and vertebral fusions^13^. Accordingly, there is some overlap between SCT and all of these conditions, with the most similarity between SCT and DA8. Consistent with this observation, one of the variants we found to underlie SCT, p.Ser243del, has been previously reported in a family with DA8^13^. Phenotypic overlap between the SCT and DA8 individuals includes mild camptodactyly, finger webbing, limited extension at the elbow, cleft palate and inguinal hernia (Table 2), but the DA8 patients are overall more severe with facial dysmorphism and pterygia. Review of the AD SCT cases described in the literature^3^ show some shared features seen in DA8 that include mild developmental delay, short stature, progressive vertebral fusions, carpal/tarsal fusions, joint immobility, fifth finger clinodactyly, hearing deficit and dysmorphic facies, though the genetic basis of disease in the published cohort has yet to be resolved. Our findings suggests that affected individuals with p.Ser243del can present clinically as either DA8 or SCT based in part on whether they presented with contractures/pytergia or progressive vertebral fusions. Neither of the other variants found to underlie SCT have been reported previously, and none of the affected individuals had major features characteristic of DA2A, DA2B, or DA8 (Table 2). Thus distinct missense mutations can lead to varying phenotypic expression, depending on the domain in which the substitution occurs and its effect on embryonic myosin function. As delineated in Fig. 3A, the p.Leu900fs9 mutation produced a truncated protein that is predicted to be deficient in the coiled coil tail domain, which provides the structural backbone to the molecule. The finding that expression of this mutation showed TGFβ signaling alterations similar to those associated with the missense and in-frame deletion mutations, differing from WT, supports the pathogenicity of the mutation. Beyond TGFβ signaling, the truncated protein may act as a loss of function or a dominant negative allele, or both.

Embryonic myosin is a cycling myosin that is expressed at high levels in the developing fetus. Its expression decreases postnatally as it is replaced, in large part, by fast myosins in postnatal and adult muscle tissue^24^,^25^. This suggests that the typical contractures found in arthrogryposis may be due to altered contractility of muscles during development, resulting in diminished fetal movement^26^. By contrast, vertebral fusions in *MYH3* SCT are progressive and the persistent embryonic myosin expression in the mutifidus muscle groups that lie between the neural arches perhaps begins to explain the progressive nature of the phenotype. Decreased canonical and noncanonical TGFβ signaling appears to be a consequence of defective embryonic myosin, and suggests a mechanism by which the progressive vertebral and carpal/tarsal fusions may result. Muscles surrounding the spine are crucial for posture and spinal support. We hypothesize that persistently abnormal function of muscles surrounding the IVD, due to the *MYH3* mutations, may result in the exposure of the disc to extraneous mechanical pressure, leading to the progressive collapse of the disc spaces over time and subsequent vertebral fusions.

These findings challenge us to understand how mutations in *MYH3* produce SCT. The altered TGFβ signaling in cells transfected with *MYH3* SCT mutations suggest that, similar to loss of FLNB, alterations in actin-interacting cytoskeletal proteins affect propagation of signaling cascades that influence tissue mechanics. Unlike SCT due to *FLNB* mutations, which directly influence the cell fate of annulus fibrosis cells, embryonic myosin SCT mutations may influence the mechanics of contractility in muscle fibers between the neural arches, indirectly affecting the annulus fibrosis. The preservation of the disc space requires a delicate balance of force and pressure and it has been shown that the spinal muscles play a central role in maintaining this balance^27^,^28^. Abnormal mechanical forces imposed upon the IVD can have a multitude of consequences including IVD deformities, calcification, and collapse^29^,^30^,^31^. We speculate that *MYH3* mutations disrupt this balance by exerting additional pressure on the disc space that eventually results in disc space collapse and vertebral fusions. Collectively, our findings illuminate a new postnatal role for MYH3 in the skeleton, particularly beyond the fetal period, and expand the spectrum of disease due to *MYH3* mutations that now includes, DA2A, DA2B, DA8 and SCT. Mechanistically, the data tie the importance of signaling and tissue responsiveness to the development of progressive vertebral fusions.

## Methods

All patients gave informed consent for this study under an approved University of California at Los Angeles Institutional Review Board human subjects protocol. All methods were approved and performed in accordance with University of California at Los Angeles Institutional Biosafety Committee’s guidelines and regulation policies.

### Histological analyses and immunohistochemistry

Tissues were fixed in 10% neutral buffered formalin, decalcified using Immunocal decalcification solution and then paraffin embedded. Paraffin blocks were sectioned sagitally at 5–10 μm, and stained. Sections used for staining were taken from the middle of the spine and each staining and IHC protocol was repeated with at least three biological replicates and three technical replicates for each biological replicate. For Hematoxylin/Eosin staining, deparaffinized and rehydrated sections were stained with Hematoxylin QS (Vector H-3404), rinsed in tap water and then destained in 0.5% Acid EtOH. Sections were then counterstained with a 0.1% Eosin Y (Sigma E4009)/90% EtOH/0.5% Glacial Acetic Acid solution.

For immunohistochemistry, paraffin sections were boiled for 20 minutes in Antigen Unmasking Solution (Vector) and subsequently stained using a Rabbit Specific HRP/DAB (ABC) Detection IHC Kit (Abcam). All experiments were performed with at least three biological replicates and four sections per replicate. Primary Antibody used for IHC: MYH3 (Genetex GTX32147).

### MYH3 plasmid Site Directed Mutagenesis

The WT MYH3 plasmid was purchased from Origine (RG218098). Site-directed mutagenesis was accomplished using the QuikChange II XL Site-Directed Mutagenesis Kit (Agilent 200521). Mutagenesis primers were designed using the QuikChange Pimer Design Program (http://www.genomics.agilent.com/primerDesignProgram.jsp). Primers used are listed in Supplementary Table 1.

### HEK cell transfection

Transfection of HEK cells was accomplished using the Lipofectamine 3000 protocol (Thermo-Fisher). Briefly, HEK cells were plated on 6-well plates at 40,000 cells/well in DMEM (GIBCO) +10% FBS (GIBCO) and left to adhere overnight. Each well was exposed to 3.75 ul of lipofectamine reagent, 1ug of plasmid DNA, and 2 ul of P3000 reagent in Opti-MEM media (GIBCO). Cells were left to incubate for two days and then serum starved for two hours before ligand stimulation.

### RT-PCR

RNA was extracted from isolated mouse IVD AF and NP as well as muscle tissue using TRIzol reagent (Life Technologies). cDNA was prepared from 1 ug of RNA using RevertAid First strand cDNA synthesis kit (Thermo Scientific) and amplified using Maxima SYBR Green/ROX qPCR Master Mix. Expression levels were calculated using the 2^deltaCT-method of analysis against the stable housekeeping gene beta-2-microglubulin (B2M) [42]. Significance was determined via Student’s T-test. Biological replicates were three times each with three technical replicates. RT-PCR primers are listed in Supplemental Table 1.

### Western blot analysis

Stimulated transfected HEK cells were rinsed with phosphate buffered saline. The monolayer cells in each well were lysed in RIPA buffer supplemented with phosphatase inhibitors (Sigma, P0044) and protease inhibitors (Sigma, P8340). Lysates were incubated at 4 °C for 30 minutes and centrifuged for 10 minutes at 10,000 rpm. The protein concentration was determined using a BCA protein assay, and equivalent amounts of protein (20 μg) were separated by electrophoresis on 10% SDS-polyacrylamide gels and transferred onto polyvinylidene fluoride membranes. After blocking for 1 hour with 5% milk in Tris-buffered saline-Tween (TBST), membranes were incubated with primary antibodies in 3% BSA/TBST solution at 4 °C with gentle shaking overnight. Membranes were incubated with horseradish peroxidase-conjugated secondary antibody at a concentration of 1:2000 at room temperature for 1 hour and detected using an ECL plus kit (Cell signaling, 7071). The band intensities were demonstrated to be in the linear range and their intensities were captured using a digital image scanner, quantified using imageJ (NIH, Bethesda, MD) and the data subjected to statistical analysis. Primary Antibodies used for Western Blots: Phospho-Smad3 (Cell Signaling, cs 9520, 1:1000), Smad3 (Cell Signaling 9523, 1:1000), Phospho-Erk p44/42 MAPK (Cell Signaling, cs 9101, 1:1000), Phospho-p38 (Cell signaling, cs 9211, 1:1000), GAPDH (Cell Signaling, cs 2118, 1:1000).

Each cell experiment was repeated with 3 biological replicates. Quantified bands were normalized to housekeeping gene levels (GAPDH). Because western blots for each biological replicate were performed separately, transfected samples were analyzed as ratios against control samples, control samples were at a value of 1. Data were analyzed by Student’s T-test; the results are shown as the mean ± standard error of a given number of trials (n) as noted in the figure legend. P ≤ 0.05 was considered statistically significant.

## Additional Information

**How to cite this article**: Zieba, J. *et al*. A postnatal role for embryonic myosin revealed by MYH3 mutations that alter TGFβ signaling and cause autosomal dominant spondylocarpotarsal synostosis. *Sci. Rep.* **7**, 41803; doi: 10.1038/srep41803 (2017).

**Publisher's note:** Springer Nature remains neutral with regard to jurisdictional claims in published maps and institutional affiliations.

## Supplementary Material

Supplementary Information

## Figures and Tables

**Figure 1:**
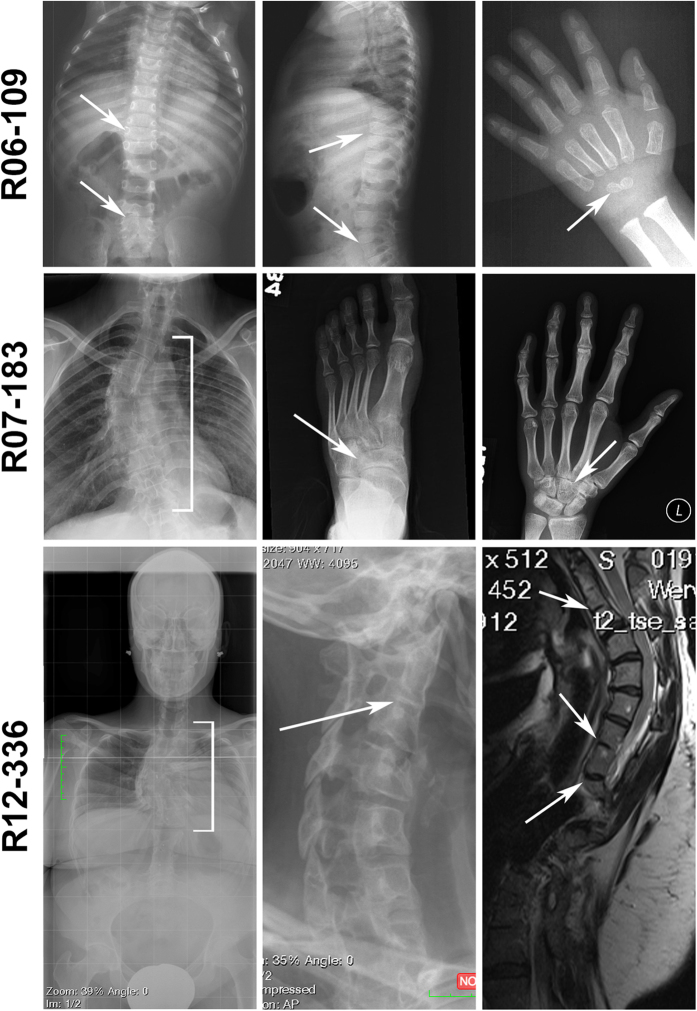
Radiographs of individuals with AD SCT. (R06-109) Three year old patient with SCT. *Left, Middle*: Radiographs of the spine showing narrowing disc spaces in the thoracic and lumbar spine (arrows). *Right*: Left hand showing developing carpal coalition (arrow). (R07-183) Adult patient with SCT. *Left*: Radiograph of the spine showing severe scoliosis and vertebral fusions in the thoracic spine (bracket). *Middle*: Left foot showing coalition of navicular and cuboid tarsal bones (arrow). *Right*: Left hand showing coalition of the hamate, trapezoid and trapezium carpal bones (arrow). (R12-336) Adult patient with SCT. *Left*: Full torso radiograph showing severe scoliosis and fusions in the thoracic spine (bracket). *Middle*: Cervical vertebrae showing narrowed disc space (arrow). *Right*: CT scan of the thoracic spine showing disc space obliteration and deformity (arrows).

**Figure 2:**
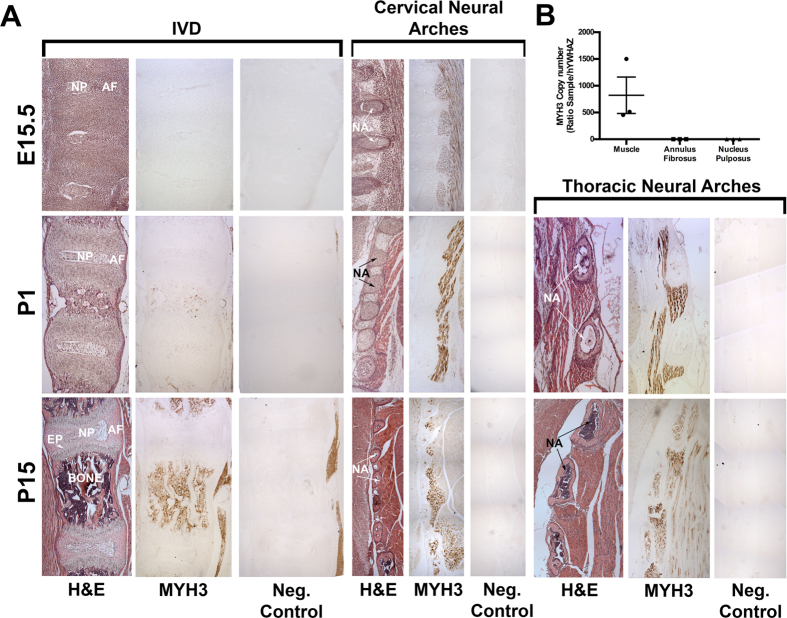
MYH3 is expressed embryonically and postnatally in muscles joining the neural arches and postnatally in bone. (**A**) Sagittal paraffin sections of spines at E15.5, P1, and P15 stained via IHC using an antibody against MYH3. N = 3 biological replicates. First column: H&E staining for morphology. Second column: IHC staining for MYH3. Third column: Negative control for IHC stain. *IVD*: MYH3 is not expressed in the developing IVD at E15.5, P1, or P15. MYH3 expression can be seen postnatally in the bone. *Cervical and Thoracic Neural Arches*: At E15.5, P1 and P15, MYH3 is expressed in the muscle between the neural arches of the cervical and thoracic spine. At P15, MYH3 expression is diminished but continues to be highly localized between the neural arches. IVD = Intervertebral Disc, NP = Nucleus Pulposus, AF = Annulus Fibrosus, NA = Neural Arch. (**B**) RT-qPCR using primers against MYH3 in skeletal muscle, the annulus fibrosus and the nucleus pulposus at P15. MYH3 is expressed in skeletal muscle but not expressed in the annulus fibrosus or the nucleus pulposus.

**Figure 3:**
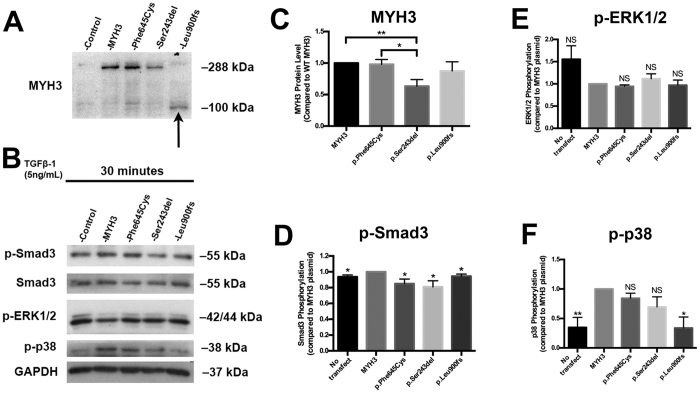
TGFβ and BMP signaling pathways are altered in cells transfected with mutated *MYH3*. (**A**) Western blot of HEK cells transfected with WT and mutant forms of the MYH3 plasmid indicating that all mutated plasmids expressed a protein. The plasmid harboring the p.Leu900fs9 mutation produced a truncated protein at ~100 kDa (arrow). (**B**) Western blot of HEK cells transfected with WT and mutant forms of the MYH3 plasmid stimulated with TGFβ-1 ligands. (**C**) Quantitation of protein stability (n = 6). (**D**–**F**) Quantitation of Western blot results normalized to GAPDH levels as well as transfected MYH3 protein levels. Because Western blots for each biological replicate were performed separately, samples transfected with the mutated plasmids or no plasmid were analyzed as ratios against samples transfected with the WT plasmid, with the WT plasmid samples set at a value of 1. Data were analyzed by Student’s T-test; the results are shown as the mean ± standard error of n = 3 biological replicates. P ≤ 0.05 was considered statistically significant.

**Table 1:** Variants identified by exome sequencing in four cases of SCT.

Individual	Chromosome	Genomic position	Reference sequence	Variant sequence	Locus	cDNA position	Protein change	Inheritance	Polyphen-2 prediction
R06-109A	17	10545582	T/T	T/G	MYH3	c.1934T > G	p.Phe645Cys	Dominant De novo	Probably Damaging
R07-183B	17	10551879	TCC	Deleted	MYH3	c.727-729delTCC	p.Ser243del	Dominant Unknown	Probably Damaging
R12-336A	17	10543102	T/T	T/del	MYH3	c.2699delT	p.Leu900fs9	Dominant Unknown	—

**Table 2:** Clinical findings in the three cases of SCT.

	R06-109A	R07-183B	R12-336A
Sex	Male	Female	Female
Familial case			
Locus	MYH3	MYH3	MYH3
Skeletal abnormalities			
Short Stature	Yes-mild	Yes-mild	Yes-mild
Cervical vertebral fusions	No	Yes	Yes
Thoracic vertebral fusions	Yes	Yes	Yes
Lumbar vertebral fusions	Yes	Yes	Yes
Sacral fusions	Yes	No	Yes
Carpal fusions	Yes	Yes	Yes
Tarsal fusions	Yes	Yes	Yes
Short stature	Yes	Yes	Yes
Cleft palate	Yes	No	No
Delayed bone age	Yes	Unknown	Unknown
Limb Abnormalities			
Camptodactyly	No	Yes-mild	No
Foot contractures	No	No	No
5^th^ digit clinodactyly	Yes	Yes	Yes
Clubbed feet	No	No	No
Limited elbow extenstion	No	Yes	No
Other Commonly Seen Clinical features in DA2A, DA2B or DA8			
Facial Contractures/Dysmorphisms	No	No	No
Microcephaly	No	No	No
Craniosynostosis	No	No	No
Cleft palate	No	Yes, family history in one individual	No
Short Neck	Yes	Yes	No
Shoulder contractures	No	No	No
Hip/Knee contractures	No	No	Torn Meniscus
Cortical thumbs	No	No	No
Webbing of fingers	No	Yes	No
Mental Retardation	No	No	No
Hyperpyrexia	No	No	No
Inguinal hernia	No	Yes, family history in one affected individual	No
Mutiple ptyergium	No	No	No
