# Supplementary material for: A postnatal role for embryonic myosin revealed by MYH3 mutations that alter TGFβ signaling and cause autosomal dominant spondylocarpotarsal synostosis
Source: Sci Rep. 2017 Feb 16;7:41803. doi: 10.1038/srep41803 (PMC5311977; doi:10.1038/srep41803)
Supplement: Supplementary Information [file srep41803-s1.pdf]

A postnatal role for embryonic myosin revealed by MYH3 mutations that alter TGFβ signaling and cause autosomal dominant spondylocarpotarsal synostosis

Jennifer Zieba, Wenjuan Zhang, Jessica X. Chong, Kimberly N. Forlenza, Jorge H. Martin, Kelly Heard, Dorothy K. Grange, Merlin G. Butler, Tjitske Kleefstra, Ralph S. Lachman, Deborah Nickerson, Michael Regnier, Daniel H. Cohn, Michael Bamshad, and Deborah Krakow

Supplementary Figure 1

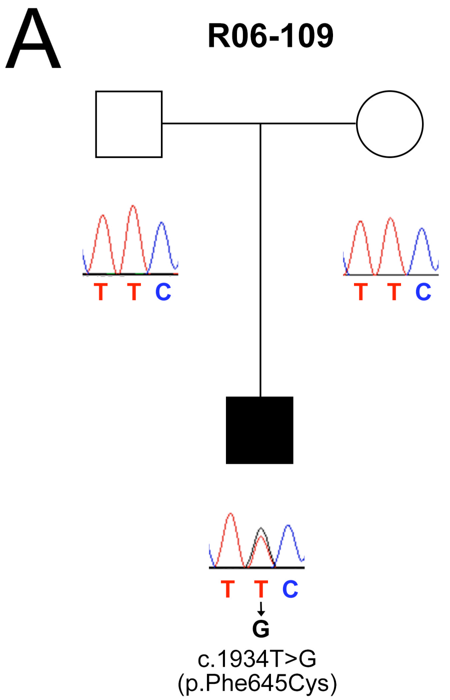

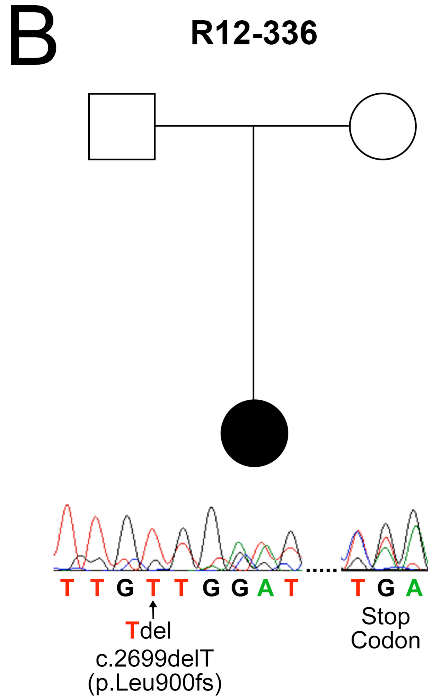

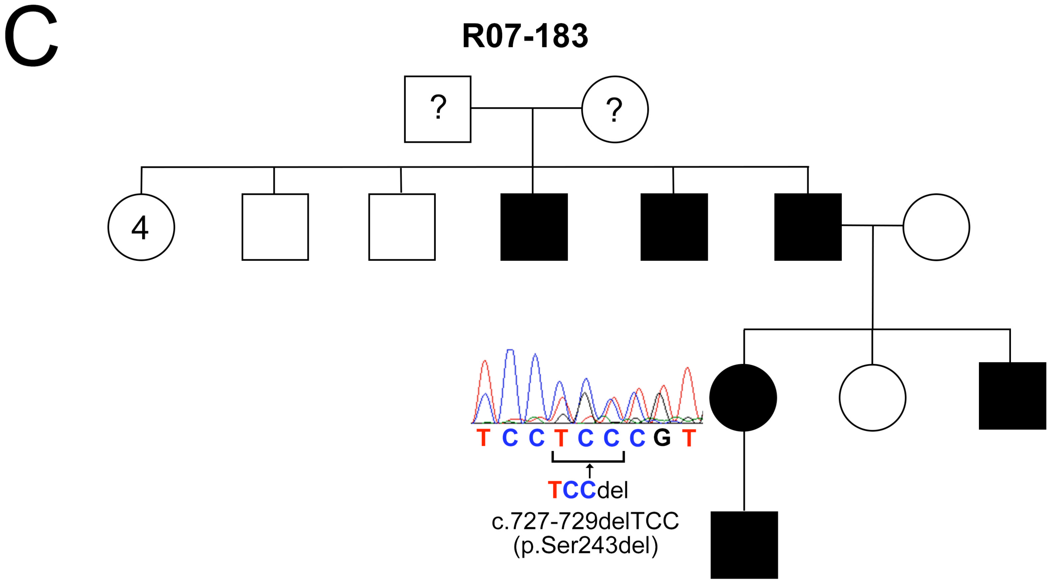

Supplementary Figure 2

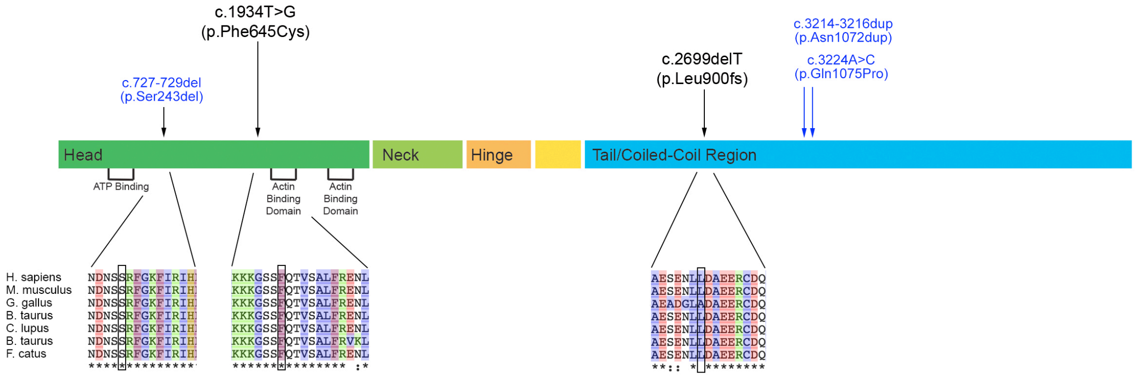

Supplementary Figure 3

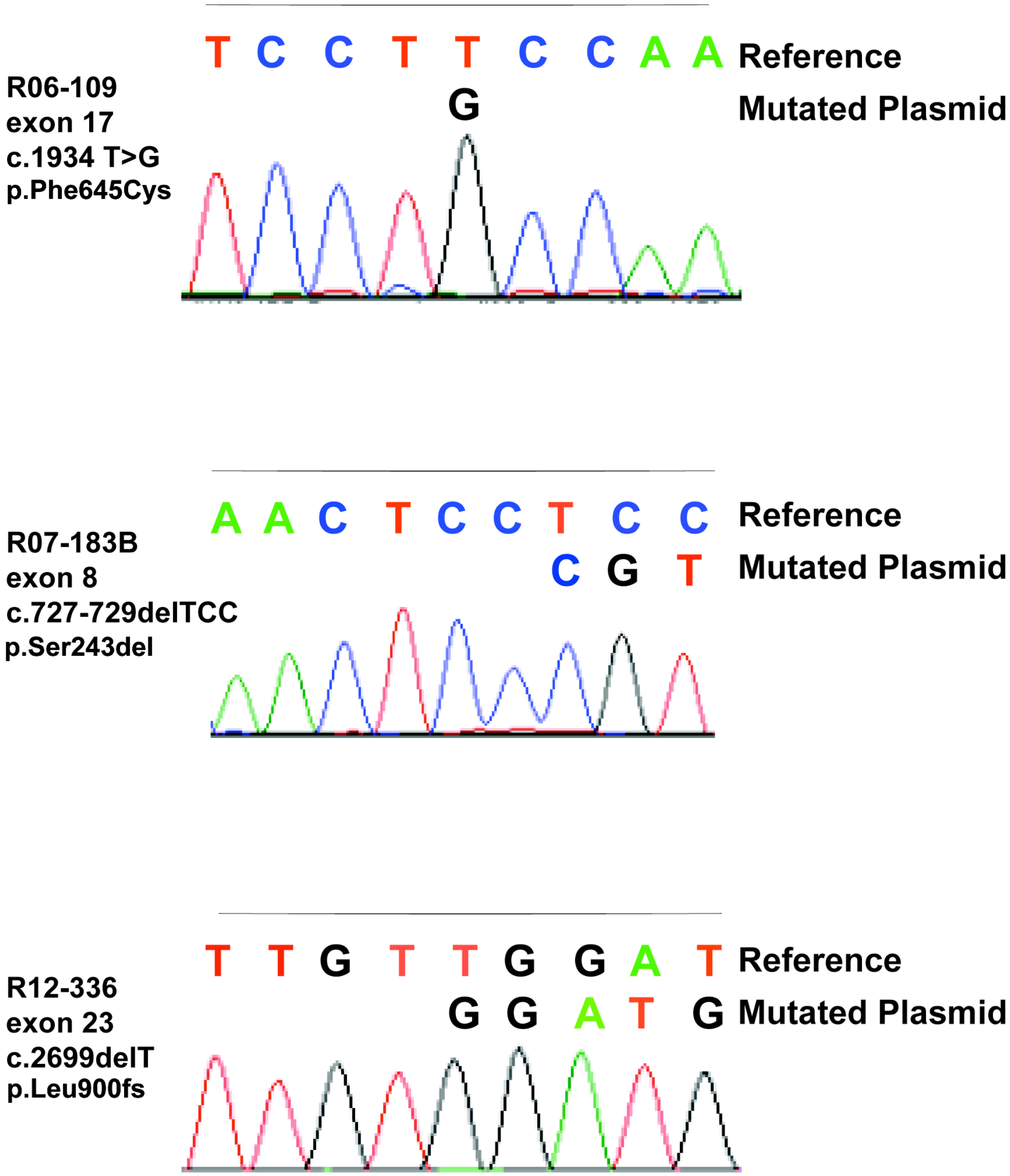

Supplemental Figure 1: Pedigree traces and Sanger sequencing confirmation of patients harboring mutations in *MYH3*. (A) R06-109 family with unaffected parents and proband heterozygous for the missense c.1934T>G mutation. Mutation is *de novo*. (B) R12-336 family has unaffected parents and the proband is heterozygous for the thymine deletion c.2699delT that results in a premature stop codon nine codons following the genetic change. (C) Three generation family (R07-183) showing autosomal dominant inheritance of the SCT phenotype. Patient with identified heterozygous c.727-729delTCC mutation in the third generation also has a son with the SCT phenotype. Phenotype of patient's grandparents is unknown.

Supplemental Figure 2: Functional domains of MYHC and locations of *MYH3* mutations. Mutations are highly conserved among species Mutations in black are found in the SCT patients. Mutations in blue have been found in DA8 patients c.727-729delTCC mutation was found in independent SCT and DA8 families.

Supplemental Figure 3: Confirmation of site-directed mutagenesis of MYH3 plasmid via Sanger sequencing. Sanger sequencing was performed to confirm mutations in each plasmid. The reference sequence is above and mutated sequence is below.

Supplemental Table 1

**Sanger sequencing primers used for human mutation confirmation**	
R06-109 Exon 17 F’	TCCATGTGATTTCCCTCCAT
Exon 17 R’	ATGCTGGAAACTCGGACAAG
R07-183 Exon 8 F’	GGAGGTGGAGGTTGCAGTAA
Exon 8 R’	TCATCATCTGTTGCCTCTGG
R12-336 Exon 23 F’	GGTGGGGATTTTTGCTTTTT
Exon 23 R’	CCTAAGAAGAATTCGCAAGCA
**Primers for site-directed mutagenesis of human MYH3 plasmid**	
R06-109 c.1934T>G Sense	GCAGAGACAGTTTGGCAGGAAGAACCCTTCTTCTT
c.1934T>G Antisense	AAGAAGAAGGGTTCTTCCTGCCAAACTGTCTCTGC
R07-183 c.727-729delTCC Sense	GTGAGGAATGACAACTCCCGTTTTGGCAAGTTCATC
c.727-729delTCC Antisense	GATGAACTTGCCAAAACGGGAGTTGTCATTCCTCAC
R12-336 c.2699delT Sense	TTCCTCAGCATCCACAAATTTTCGCTTTCAGCTTGTAC
c.2699delT Antisense	GTACAAGCTGAAAGCGAAAATTTGTGGATGCTGAGGAA
**Primers used to confirm mutations in human MYH3 plasmid**	
R06-109 c.1934T>G F’	TCACTGATCCACTATGCGGG
c.1934T>G R’	TAGCCCCTGGAGTTTTGGTT
R07-183 c.727-729delTCC F’	CTCCATCTCTGACAACGCCT
c.727-729delTCC R’	GCTTCCCAGTGGTTCCAAAA
R12-336 c.2699delT F’	AAGTCGGAGGCAAAGAGGAA
c.2699delT R’	TGGCATGCTTCTCCTTCTCA
**RT-PCR primers against mouse MYH3**	
Forward Primer	GGATCGAAGCTCAGAACCAG
Reverse Primer	AGGACGTGTATCGGTCCTTG
